# Discoidin Domain Receptors in Melanoma: Potential Therapeutic Targets to Overcome MAPK Inhibitor Resistance

**DOI:** 10.3389/fonc.2020.01748

**Published:** 2020-09-11

**Authors:** Coralie Reger de Moura, Marco Prunotto, Anjum Sohail, Maxime Battistella, Fanelie Jouenne, Daniel Marbach, Celeste Lebbé, Rafael Fridman, Samia Mourah

**Affiliations:** ^1^Laboratory of Pharmacogenomics, Hôpital Saint-Louis, AP-HP, Paris, France; ^2^INSERM, UMR_S976, Université de Paris, Paris, France; ^3^School of Pharmaceutical Sciences, University of Geneva, Geneva, Switzerland; ^4^Department of Pathology, School of Medicine, Karmanos Cancer Institute, Wayne State University, Detroit, MI, United States; ^5^Department of Pathology, Hôpital Saint-Louis, AP-HP, Paris, France; ^6^Roche Pharmaceutical Research and Early Development, Pharmaceutical Sciences, Roche Innovation Center Basel, F. Hoffmann-La Roche Ltd., Basel, Switzerland; ^7^Department of Dermatology, Hôpital Saint-Louis, AP-HP, Paris, France

**Keywords:** DDR1, melanoma, drug resistance, MAPK inhibitors, therapeutic target

## Abstract

Melanoma is a highly malignant skin cancer with high propensity to metastasize and develop drug resistance, making it a difficult cancer to treat. Current therapies targeting BRAF (V600) mutations are initially effective, but eventually tumors overcome drug sensitivity and reoccur. This process is accomplished in part by reactivating alternate signaling networks that reinstate melanoma proliferative and survival capacity, mostly through reprogramming of receptor tyrosine kinase (RTK) signaling. Evidence indicates that the discoidin domain receptors (DDRs), a set of RTKs that signal in response to collagen, are part of the kinome network that confer drug resistance. We previously reported that DDR1 is expressed in melanomas, where it can promote tumor malignancy in mouse models of melanoma, and thus, DDR1 could be a promising target to overcome drug resistance. In this review, we summarize the current knowledge on DDRs in melanoma and their implication for therapy, with emphasis in resistance to MAPK inhibitors.

## DDR1, a Worse Prognostic Biomarker and an Emerging Target

Among the receptor families known to mediate the interaction of cells with collagen, the discoidin domain receptors (DDRs) constitute a major class. The DDRs are RTKs which undergo activation upon binding to collagens. There are two members in the DDR family, DDR1 and DDR2, with DDR1 comprising 5 isoforms, two of which are inactive or truncated receptors. There is only one DDR2 isoform. Structurally, full-length DDRs are multidomain type I transmembrane glycoproteins, comprising an extracellular discoidin domain, a transmembrane region, and an intracellular segment that includes a kinase domain [for structural details of DDRs see (1, 2)]. The reason for diversity in DDR1 isoforms is still unknown, but their structural differences may be necessary to activate distinct signaling pathways. The ability of DDRs to recognize collagens as ligands places these receptors in a unique category among the RTK family because the collagen family is comprised of 28 distinct members with different structural organizations, biomechanical properties, and tissue distributions (3). To recognize and respond to the various members of the collagen superfamily under various conditions and in different tissue locations, DDRs become versatile kinases, able to interact with distinct collagen types and initiate the downstream pathway in response to alterations on collagen properties in diverse physiological and pathological conditions. DDRs undergo receptor autophosphorylation in response to both fibril- and network-forming collagens. For instance, DDR1 and DDR2 are activated by several fibrillar collagens, albeit with different efficiencies. However, both receptors are efficiently activated by the ubiquitous fibrillar collagen type I (2, 4–6). In contrast, DDRs differentially respond to the network-forming collagen IV and X, with DDR1 being activated by collagen IV while DDR2 by collagen X (6). The ability of DDRs to recognize distinct collagen types has significant implications in conditions in which cells traffic through different tissue compartments. In cancer, for instance, premalignant and fully malignant carcinoma cells can express DDR1. Thus, as cells progress from normalcy to malignancy and acquire the ability to invade basement membranes (BM) and the underlying stromal matrix, the expression of DDR1 may modulate cancer cell behavior in response to both collagen IV and collagen I, possibly by initiating ligand-specific signaling pathways. On the other hand, DDR2, which is not usually expressed in epithelial cells, has been shown to be induced during the process of epithelial-to-mesenchymal transition (EMT), a molecular and cellular program that has been associated with enhanced invasive capacity (7, 8). Thus, DDR2 together with DDR1 may contribute to the activation of signaling pathways associated with interactions of carcinoma cells with both network-forming and fibrillar collagens, as they traffic through various matrix compartments. Although DDRs are implicated in normal organ development and function (2), there is multiple evidence showing that DDRs are critical players in cancer progression, regulating multiple aspects of malignancy including cell proliferation, migration, invasion, and drug resistance (9, 10). These effects of DDRs on malignant cell behavior appear to be mediated mostly via collagen-dependent receptor phosphorylation; however, evidence has shown that DDRs can also elicit pro-malignant activities in a kinase-independent manner (11, 12). In this regard, these studies highlight the importance DDR–collagen interaction through the discoidin domain, independent of kinase activity, in mediating the functions of DDRs in cells. However, more data are needed to establish a clear distinction between collagen-independent and -dependent effects of DDRs in cancer progression. While there is consensus on the pro-malignant effects of DDR2 in cancer, this is not the case for DDR1. Indeed, evidence suggests that DDR1 can elicit either tumor-promoting or -suppressing effects on cancer in a context-dependent manner [reviewed in (13)], possibly due to the fact that DDR1 plays a role in the maintenance of normal epithelial integrity by regulating cell–BM and cell–cell interactions (14–17). On the other hand, many studies have shown that overexpression of DDR1 in several cancer types correlates with disease progression (18–20). However, it is important to note that expression analyses in tissue samples are limited because a pro-malignant role for any gene cannot be asserted without functional studies. Regardless, the emerging picture for DDR1’s role in cancer progression is complex, likely involving tumor-suppressive and/or promotive effects. In this review, we will focus on DDR1 and melanoma, and its potential role in promoting malignant features and as a potential target to overcome drug resistance.

## Melanoma-Targeted Therapies and Resistance

Over the past few years, numerous therapies have emerged in the management of advanced melanoma, which have profoundly transformed the therapeutic landscape and prognosis of this disease. Drug development has been driven by the unveiling of the molecular characteristics of melanomas, which provided new insights into the signaling networks that are operative in this disease (21). The mitogen-activated protein kinase (MAPK) pathway was found to be dysregulated in a significant proportion of melanomas. This dysregulation is mostly caused by the fact that the majority of melanomas harbor a mutation on the serine–threonine kinase BRAF (V600), which is part of the MAPK signaling pathway. Overall, over 90% of BRAFV600-mutated melanomas harbor a BRAFV600E mutation, 6% a BRAFV600R mutation, and 4% a BRAFV600E2, BRAFV600D, or a BRAFV600K mutation (22), and therefore mutated BRAF kinase became an attractive therapeutic target (23). As a result, several inhibitors (vemurafenib, dabrafenib, and encorafenib) were developed, which improved survival of melanoma patients when compared to conventional chemotherapy (24–26). Almost at the same time, inhibitors of MEK, a downstream signaling kinase of the MAPK pathway, were developed. These compounds (trametinib, cobimetinib, and binimetinib) also exhibited significant activity in BRAFV600-mutant melanoma (27, 28). Clinical trials evaluating the combination of BRAF inhibitors (BRAFi) with MEK inhibitors (MEKi) demonstrated significant clinical efficacy, as indicated by improved overall survival (OS) and progression-free survival (PFS). These promising results led to the approval of dabrafenib/trametinib and vemurafenib/cobimetinib combinations for patients with advanced, metastatic BRAFV600-mutant melanoma (29–31).

Despite these advances in melanoma treatment, acquired resistance to MAPK-targeted therapy is almost inescapable (32), and, as expected, resistance to BRAFi and MEKi was also found in melanoma patients. The mechanisms of resistance to MAPK inhibition are multiple and complex. In many cases, resistance is caused by reactivation of the MAPK pathway (RAS mutation, MEK and/or BRAF amplification, differential splicing leading to truncated variants of BRAF, activation of MAPKK), activation of the PI3K pathway through genetic alterations of PTEN, overexpression and activation of PDGF, IGF1, or c-Met receptors, or development of a pro-oncogenic tumor microenvironment (33–36). Another mechanism of resistance involves the action of ERBB3, a member of the EGF family of receptors, which is known to be over-expressed in human melanoma. Evidence has shown that BRAFi and MEKi therapeutic effects on BRAFV600-treated tumors are decreased by enhanced ERBB3 signaling, suggesting that ERBB3 is implicated in adaptive resistance to BRAF and MEK inhibitors. These observation suggested that a combination of ERBB2/EGFR inhibitor, which block NRG1/ERBB3 signaling, with BRAF and MEK inhibitors could overcome resistance (37, 38). It is worth mentioning that, eventually, these multiple and distinct mechanisms of resistance to MAPK inhibitors result in ERK reactivation, demonstrating the extent to which melanoma cells are addicted to ERK signaling for proliferation and/or survival (36). Recently, characterization of tumor cell and stromal/immune transcriptomic alterations in MAPKi-treated melanomas provided insight into the responses elicited by these inhibitors, even at early stages of treatment (39). Song et al. showed that an immune-phenotypic transition due to MAPK-targeted therapies could involve a loss of T-cell inflammation leading to an anti-PD1 resistance in melanoma, even at early stages of treatment. These studies suggested that several adaptive responses in both the tumor (intrinsic) and the immune system (extrinsic) are operative, which could offer new therapeutic opportunities to overcome resistance. The studies of Yan et al. also demonstrated that BRAF and MEK inhibitor-treated patients, showing complete responses, have preexisting tumor immunity transcriptomic signatures that are higher than those expressed in patients with progressive disease, suggesting that enriched immune infiltration improves response to BRAFi and MEKi combination (40). These observations highlight the crucial need for a better understanding of treatment effects on both the tumor and its microenvironment but also for more effective therapies aimed at overcoming or preventing drug resistance in melanoma patients. Several alternative strategies, including paradox breaker RAF inhibitors and ERK inhibitors, are currently under investigation in the BRAFi + MEKi resistance setting (41, 42). In this context, the validation of new and promising targets is the cornerstone of this challenge. Because DDRs are crucial regulators of tumor cell behavior in response to their immediate microenvironment and in light of our recent data on DDR1 in melanoma (43), we propose that DDR1 targeting in melanomas resistant to MAPK inhibitors is worth exploring.

## DDR1 in Melanoma

Melanomas are derived from melanocytes, the melanin-producing cells in the epidermis. Melanocytes are located in the basal layer of the epidermis, making contact with the BM (44). Previous evidence demonstrated a role for DDR1, a major collagen IV receptor, in mediating the interaction of melanocytes with the BM. Adhesion of melanocytes to collagen IV, induced by overexpression of the matricellular protein CCN3, was mediated by upregulation of DDR1 protein expression, and silencing of DDR1 mRNA reduced CCN3-induced adhesion to collagen IV (45). However, whether this adhesive effect of DDR1 was mediated via its kinase activity was not determined. Regardless, CCN3-mediated DDR1 upregulation was proposed to play a major role in the adhesion of melanocytes to the BM and in the maintenance of skin homeostasis (45). While these *in vitro* studies suggested a role for DDR1 in melanocytes in normal skin, our recent immunohistochemical analyses in human skin sections demonstrated that DDR1 immunoreactivity was only detected in normal keratinocytes, albeit at relatively low levels of expression (43). Moreover, we found no detectable DDR1 expression in benign naevi in all cell types. Analyses of skin samples harboring melanoma showed a strong expression of DDR1 in the melanoma cells, which was positively correlated with invasive depth and patient survival. Our functional *in vitro* studies also showed a key role for DDR1 in melanoma cell proliferation, migration, invasion, and survival (43). Importantly, a pan-DDR inhibitor, DDR1-IN-1 (46), with higher selectivity toward DDR1 than to DDR2, decreased tumor growth in *BRAF*-mutated human melanoma xenograft models (43). Because melanoma and stromal cells also express DDR2, these preclinical studies with DDR1-IN-1 suggest that DDR1, and possibly DDR2, constitutes a potentially new target in melanoma (43). Based on these results, we posit that DDRs are promising therapeutic targets in *BRAF*-mutated melanomas.

**FIGURE 1:**
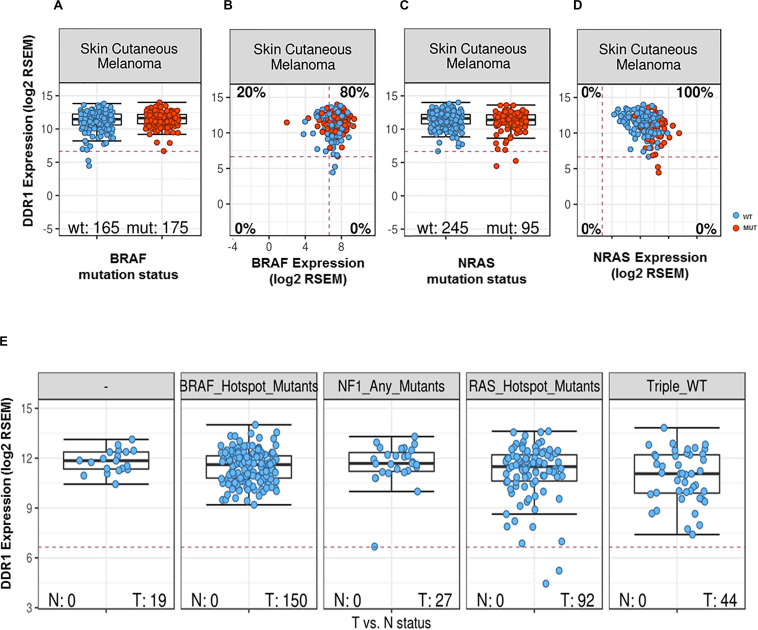
DDR1 expression in melanoma samples as a function of expression and mutational status of *BRAF* **(A,B)** or *NRAS* **(C,D)**. Transcript abundances were quantified using RSEM (50) on a log2 scale. The red dashed lines indicate the typical cutoff for expressed genes [RSEM = log2(100)]: samples below the red lines are assumed to have no or very low expression. Classification of melanoma subtypes into mutant BRAF, mutant RAS, mutant NF1, and triple-WT (wild-type) was obtained from TCGA (51). **(A)** DDR1 expression for BRAF wild-type (left, blue) and mutated cases (right, red). **(B)** DDR1 versus BRAF expression for WT (blue) and BRAF-mutated cases (red). 80% of the samples show co-expression of DDR1 and BRAF (upper-right quadrant). **(C)** DDR1 expression for NRAS wild-type (left, blue) and mutated cases (right, red). **(D)** DDR1 versus NRAS expression for wild-type (blue) and cases with NRAS mutations (red). **(E)** DDR1 expression in melanoma molecular subtypes (BRAF, NRAS, NF1 mutated, and triple WT). There is no significant difference in the median expression of DDR1 (*p*-value > 0.05, Wilcoxon rank-sum test), but we note some outliers with low DDR1 expression in the NF1 mutant, RAS mutant, and triple WT cases (three left-most plots), while no outliers with low expression are observed for the BRAF mutant subtype. N, normal; T, tumor.

To further examine the association between DDR1 and melanoma, we analyzed a curated set of seven non-redundant cutaneous melanoma cohorts from the cBioPortal site (47, 48). Out of a total of 667 patients, 114 (10.6%) were identified as harboring genetic alterations in DDR1. However, although the mutational burden in melanoma is higher compared to other types of cancers, no difference in survival was observed in patients harboring mutated DDR1. Analyses of TCGA database samples for DDR1 expression vs. BRAF mutational status showed DDR1 to be slightly upregulated in BRAF-mutated cancers (effect size 0.16, *p* = 0.00031; differential expression analysis of mutated vs. wild-type cases using a linear model) and with a similar tendency, but not statistically significant, between WT and mutated BRAF melanoma samples (effect size 0.27, *p* = 0.061, Figure 1A). However, we found that DDR1 and BRAF are co-expressed in the majority of skin melanoma samples (80%) regardless of BRAF mutational status (Figure 1B). Analysis of the same database for DDR1 expression vs. NRAS mutational status showed DDR1 to be slightly downregulated in all NRAS-mutated cancers (effect size −0.26, *p* = 0.00053) and with a similar tendency (effect size −0.29, *p* = 0.065, Figure 1C). As with BRAF, DDR1 and NRAS are co-expressed in almost all samples, regardless of NRAS mutational status (Figure 1D). Although not statistically significant, the analysis of the different skin melanoma subtypes showed that DDR1 expression is always high for BRAF and NF1 mutants, while there are few outliers with low DDR1 expression for RAS mutants and triple wild-type samples (Figure 1E).

## Conclusion

The emerging evidence suggests that DDRs are part of the signaling networks that contribute to melanoma progression. However, more studies are warranted to dissect the molecular mechanisms by which DDR-initiated signaling influences melanoma cell behavior. Melanomas are also characterized by a stroma rich in collagen (49), which constitutes a barrier for invading tumor cells but may also actively promote disease progression through DDR signaling. We posit that a DDR/collagen axis may contribute to the resistant phenotype of BRAF-mutated melanomas and therefore a rationale target to restore therapeutic efficacy.

## Author Contributions

CR, MP, AS, RF, and SM contributed to development, writing, and final review of the article. MP and DM contributed to the database analysis. MB, FJ, DM, and CL contributed to the final review of the article. All authors contributed to the article and approved the submitted version.

## Conflict of Interest

DM is employed by Roche, MP is an employee of Galapagos Ltd., at 80% employment rate, this work was performed during his 20% academic time at the University of Geneva. MB declares a consulting role for Histalim, BMS, and Innate Pharma. CL declares honoraria from Roche, advisory roles at Roche, GSK, Novartis, BMS, MSD, and Amgen and travel accommodation provided by Roche. SM declares a consulting role at Roche, Janssen, Biocartis and Novartis. The remaining authors declare that the research was conducted in the absence of any commercial or financial relationships that could be construed as a potential conflict of interest.
